# Obituary

**Published:** 1851-02

**Authors:** Clinton Colegrove


OBITUARY. *To the Editor of the Buffalo Medical Journal:—*

DEAR SIR,—Hardly has there occurred to me a more earnest admonition of the uncertainty of life, than the recent demise of an intimate and noble friend and companion of my pupilage, Alfred Milton Rowe. A year ago, of the one hundred and fifteen disciples who frequented the amphitheatres of the Medical College at Buffalo, none with stricter fidelity fulfilled the obligations of a sincere courtier of useful knowledge, or more assiduously aspired at a career of discipline and distinction. You can not fail to remember the sincerity and dignity of his demeanor, and the air of severe devotion to the duty of preparation for professional labor, which he manifested in the lecture room. As a companion, he was cordial, constant, fraternal. A more congenial and cheerful associate, I have seldom met. His heart was a fountain of sympathy and disinterested sentiment. He had eminently the characteristics and impulses of a broad humanity.

The features of his mental constitution were by no means so ordinary as not to justify the most gratifying hopes of success as a practitioner and medical inquirer. He had a readiness of suggestion and originality of argument, which rendered him if not formidable, at least much to be respected in private debate; and he so ably mustered the expository lore of the amphitheatre, as to match and confound the theoretic elasticity of his antagonists.

In allusion to the means of improvement, and the pleasures of social intercourse, which he so well appreciated and enjoyed, he often, as if involuntarily, exclaimed—"These are golden days!" Truly, with what a feeling of exhilaration and hope did he glance over the field of prospective industry and usefulness; and how suddenly has the earthly vision fled!

The tide of life, the mystery of whose perpetuity, and the phenomena of whose derangements he was struggling to comprehend—ere his work was accomplished, was arrested in his own stature, and the spirit which animated forsook the tabernacle in which he sojourned. Touched by the remembrance of his fidelity and promise, his personal and peculiar excellence, his industry and his endowments, I can not withhold so brief a recapitulation of virtues not less worthy of imitation than commemoration.

Let the living profit by his ambition, of which the sum is thus expressed:

Si honorem, non laborem,
Quaeris, frustra niteris,
Si prae-esse, non prodesse,
Studes, nihil efficis.

And as well ought it to be remembered, that the little island of existence alloted to mortals, but briefly overlies the infinite sea into whose unexplored profound we all must come, but in whose solemn solitudes are the portals of eternal life to the wise.

I append a short paragraph from the letter of a friend of the deceased:

"Mr. Rowe left Gowanda, where he had previously resided, in May, 1850, and went to Glade Mills, Butler Co., Pa., where he located himself in business, and designed to remain for some time. He had been there but a few weeks, when he was attacked with bilious fever, and died Sept. 13th, after an illness of four weeks. His friends were not acquainted with his sickness, until a week before his death, when he expressed a wish to see his father. A letter was despatched without delay, but too late. He was buried before his father's arrival. His remains were taken to Sunderderland, Mass., where his family and friends reside. He was perfectly conscious to the last, and seemed prepared for the great change that awaited him. His age was 24."

Respectfully yours,
CLINTON COLEGROVE.

